# Myeloma: Patient accounts of their pathways to diagnosis

**DOI:** 10.1371/journal.pone.0194788

**Published:** 2018-04-04

**Authors:** Debra A. Howell, Ruth I. Hart, Alexandra G. Smith, Una Macleod, Russell Patmore, Gordon Cook, Eve Roman

**Affiliations:** 1 Epidemiology & Cancer Statistics Group, Department of Health Sciences, University of York, York, United Kingdom; 2 The Hull York Medical School, University of Hull, Hull, United Kingdom; 3 Department of Haematology, Queen’s Centre for Oncology and Haematology, Castle Hill Hospital, Cottingham, Hull, United Kingdom; 4 Department of Haematology, St. James’s Institute of Oncology, St James’s University Hospital, Leeds, United Kingdom; University of Stirling, UNITED KINGDOM

## Abstract

**Background:**

Pathways to myeloma diagnosis can be prolonged, and are often preceded by multiple GP consultations and emergency presentation. This is the first qualitative study to examine events leading to diagnosis by asking patients about their experiences during this time.

**Methods:**

Set within a UK population-based cohort, semi-structured interviews were conducted with 20 myeloma patients with varying characteristics and pathways, 12 of whom invited their relatives to take part. Interviews were audio-recorded and qualitative analysis undertaken.

**Results:**

Pre-diagnostic awareness of myeloma was minimal. Disease onset was typically described as gradual, and health changes vague but progressive, with increasing loss of function. A wide range of symptoms was reported, with the similarity of these to self-limiting conditions failing to raise suspicion of myeloma among patients and GPs. Patients tended to normalise symptoms at first, although all eventually sought GP advice. GPs often initially suggested benign diagnoses, which were sometimes only revised after multiple consultations with persistent/worsening symptoms. Referrals were made to various hospital specialities, and haematology if associated with abnormal blood tests suggestive of myeloma. Once in secondary care, progress towards diagnosis was generally rapid.

**Conclusions:**

Accounts confirmed that pathways to diagnosis could be difficult, largely due to the way myeloma presents, and how symptoms are interpreted and managed by patients and GPs. Recognition of ‘normal’ health and consultation patterns for the individual could promote appropriate help-seeking and timely referral when changes occur, and may be more effective than raising awareness about the myriad of potential symptoms associated with this disease.

## Background

Evidence suggests that pathways leading to myeloma diagnosis can be difficult. Compared to patients with other cancers, those with myeloma are the more likely to have multiple GP consultations before hospital referral and present via an emergency route; with such factors resulting in prolonged times to diagnosis [1–3]. This is concerning as these patterns tend to be associated with more advanced disease, and poorer quality of life and survival [1,4–6]. For well over a decade, early diagnosis has been promoted in the UK through policy, targets and guidance [7,8]. Whilst the latter has reduced the average time to diagnosis of some cancers, this is not so for myeloma, where the interval between first presentation with symptoms in primary care and diagnosis has in fact increased [3].

With an estimated 4,300 new diagnoses annually in the UK, myeloma is a relatively rare disease; median age of onset is around 73 years and it is more common among males than females [9]. Existing UK research exploring events before diagnosis largely examines time-intervals, derived from nationally collected routine data or large surveys [1,10,11]. There is currently no published qualitative evidence from patients about their experiences during this time. The present study addresses this deficit, exploring the experiences of patients’ (and their relatives’) in the time leading to diagnosis, their perceptions of whether delays occurred, and if so, why. We focus on the time from detection of a health change to first help-seeking with a healthcare practitioner (the appraisal and help-seeking intervals), and the time from first help-seeking to diagnosis (the diagnostic interval) [12].

## Methods

We conducted a qualitative study [13], undertaking in depth interviews to generate rich narratives from patients and their relatives, which were analysed to provide insight and understanding about how participants made sense of their pathways to diagnosis.

### Context

The study is nested within the Haematological Malignancy Research Network (HMRN: www.hmrn.org), a UK population-based cohort instigated in 2004 to generate ‘real world’ data for research and clinical purposes [9,14–16]. Briefly, HMRN includes all patients newly diagnosed with a haematological malignancy in the study area (~2,200 annually from a population of around four million people that is representative of the UK as a whole). Clinical care within HMRN adheres to national guidance, and as part of routine practice a core dataset (prognostic, full treatment and outcome) is abstracted from all patients’ medical records. Participants in this sub-study are derived from within HMRN; and detailed methods are described below, in accordance with the consolidated criteria for reporting qualitative studies (COREQ) [17].

### Theoretical framework

The study methodology is based on qualitative description, a pragmatic, naturalistic approach which focuses on producing ‘a comprehensive summary of events in the everyday terms of those events’ [18,19]. Qualitative description aims to produce ‘straight descriptions’ and ‘minimally theorized’ findings with practical applications, using a range of techniques including those associated with methodologies such as grounded theory (20) and constructivist grounded theory [20,21]. The study was also informed by existing models of help-seeking behaviour and care pathways [12,22,23].

### Participant selection

Patients were eligible for inclusion if they were newly diagnosed with myeloma within the HMRN area and aged 18 years and above; they were excluded if they were aged under 18 years, or did not have the capacity to give informed consent, as verified by their clinical team. HMRN patients are routinely invited to complete a questionnaire about their symptoms and help-seeking, usually within four to six weeks of diagnosis, and participants were selected from those returning this information. Most were diagnosed with myeloma between April and November 2015, the exception being two early pilot interviews.

Sampling was purposive, targeting a range of patients, from those who described difficult or prolonged experiences, to those who appeared to have less complex, shorter pathways. We recruited across both sexes and a range of ages. Of 34 patients invited to participate, 20 agreed and were interviewed (14 men and 6 women, aged 43–78 years, with prolonged and shorter pathways), with 12 inviting a relative to join the discussion. All were interviewed within a year of diagnosis.

### Data collection

Potential participants were posted a study pack containing a letter, information leaflet, response form, and prepaid return envelope. Those wishing to participate contacted the study team directly, and an interview was arranged. Patients were recruited and interviews conducted largely between November 2015 and May 2016 by one of two researchers (RH/DH), neither of whom were known to participants; most took place at the patient’s home, although two opted for a telephone discussion.

Interviews were semi-structured, meaning the discussion was guided by a schedule spanning time from first symptom(s) to diagnosis (Box 1). The schedule was developed from existing literature and clinical expertise within the study team. It denoted issues considered important to explore during each discussion, but was flexible enough to be adapted in situ to take account of the experiences, activities and events perceived as significant by individual participants.

**Box 1: Summary interview schedule**

- Initial/subsequent health changes/symptoms
- Interpretation of health changes/symptoms (by patients/healthcare professionals)
- Decision to seek help
- Factors encouraging/discouraging help-seeking
- People/places where help sought
- Reappraisal of health changes/symptoms (by patients/healthcare professionals)
- Perspectives about time taken to diagnose myeloma
- What worked well/could be improved
- Prior knowledge/experience of myeloma
- Anything else considered relevant

Each interview lasted around 45 minutes, was audio-recorded, transcribed verbatim, checked and anonymised. Field notes, made after each discussion, were used to support the transcripts. Data collection was discontinued once ‘saturation’ was reached. This is the point at which no new issues or themes emerge, suggesting data are sufficient to support insightful analysis and sampling may be redirected and/or discontinued [20].

### Data analysis

Analysis was iterative, running alongside and informing data collection (RH). After familiarisation with transcripts, several rounds of coding were undertaken, with refinement driven by constant comparison. Memoing and mapping techniques [21] were used to draw out patterns (e.g. similarities and differences between accounts) and relationships (e.g. between codes). Pathway maps, and preliminary findings from the data analysis were discussed with the wider study team (DH, ER and AS), assessed, and modified as necessary. Time intervals (from symptom onset to first help-seeking, and first help-seeking to diagnosis) were estimated from dates provided by patients during interviews and in the HMRN routine questionnaire about symptoms and help-seeking.

### Ethical approval

Ethical approval was obtained from the Yorkshire & Humber National Research Ethics Service (REC: 12/YH/0149) and all patients gave written consent.

## Results

The pathways described generally demonstrated a complex interplay between patients and GPs over time. This process was not typically linear (i.e. moving in one direction from appraisal, to help-seeking, then diagnosis), but often involved patients appraising and reappraising health changes and symptoms as their disease progressed and seeking help on multiple occasions.

Reflective of our intent to include people with varying experiences, time to diagnosis differed markedly between patients (Table 1). Based on estimated dates provided by participants, the time from initial health change to first help-seeking with a healthcare practitioner ranged from around 1 to 7 months, and from help-seeking to diagnosis it was 2 weeks to 17 months. Patients reported between one and ten primary care consultations with what they considered (in hindsight) to be myeloma symptoms, before the referral leading to diagnosis. Twelve of the 20 patients considered their diagnosis delayed: *‘I just think*, *you know*, *they maybe could have found it earlier’* [Patient (P) 15]. Correlations between ‘delay’ and time to diagnosis varied, however, with a patient diagnosed in under four months of the initial health change perceiving delay, whilst another diagnosed after around a year did not.

**Table 1:** Characteristics of interviewed myeloma patients (n = 20), health changes/symptoms and pathway to diagnosis.

Patient ID	Sex	Age (Yrs)	Health changes/symptoms reported prior to myeloma diagnosis	Healthcare professionals consulted^1^ and secondary care referrer (R)^2^	Referral specialities consulted prior to diagnosis^1^^,^^3^	Primary care consultations with myeloma symptoms^1^	Estimated duration of intervals (months)		Patient perceived delay
							Appraisal & help-seeking	Diagnostic	
**P01**	M	67	Under-performance^4^, trouble breathing, pain (shoulder blades), tiredness.	GP Practice nurse (R1)	**R1-Respiratory medicine**^5^ • Haematology	6	1	2.5	Yes
P02*	M	63	Heartburn/indigestion, pain (chest, armpits, arms), under-performance, tiredness, poor sleep (due to pain), visible lump (sternum), pale.	GP (R1)	**R1-Rheumatology** • Haematology • Oncology	3	2.5	7	Yes
**P03**	M	71	Irritable/restless knee (post-replacement), under-performance, pain (back, ribs–later identified as fractures), cramp, abnormal blood test (blood ‘*a bit thick*’).	GP (R1) Physiotherapist (x 2 –one new; one ongoing due to knee replacement–x-ray normal)	**R1-Haematology**	6	1	17	Yes
**P04**	F	78	Consulted regarding discomfort associated with existing condition (hernia). Abnormal blood test (‘*rather anaemic*’), tiredness, under-performance, shivers.	GP (R1 & R2)	**R1-Gastroenterology** (endoscopy and colonoscopy) **R2-Haematology**	3	n/a (existing condition)	5	Yes
**P05**	F	55	Tiredness, pain (chest, ribs, back), nosebleeds, infection (persistent sore throat), abnormal blood test (‘*severely anaemic… immune system shot to bits*’).	GP (R1) Locum GP (requested bloods/x-ray)	**R1-ENT** (GP approves patient’s decision not to attend due to improvement) **2-Haematology** (hospital re-called patient–abnormal blood test)	4	1	4	No
**P06**	F	71	Nausea, sickness (eventually intolerant of sips of water), oral thrush, couldn’t eat, weight loss, abnormal blood test (‘*severely dehydrated*’).	Chemist GP (R1)	**R1-Gastroenterology** (endoscopy) • Acute medical unit (emergency admission due to blood test result) • Renal medicine • Haematology	2	1	1.5	No
**P07**	F	56	Recurrent mouth ulcers, abnormal blood test (‘*folic acid deficiency*’), under-performance, breathless, dizzy, palpitations, ‘run-down’, abnormal blood test (‘*everything below range*’), abnormal urine test (‘*Bence Jones positive’*).	GP Nurse practitioner (R1)	**R1-Haematology** (urgent)	5	3	8	Yes
**P08**	M	68	‘Flu’ symptoms (diagnosed as pneumonia); abnormal blood test (unknown what).	GP (R1)	**R1-Acute medical unit** (emergency admission via GP) • High dependency unit • Haematology (patient called in–abnormal bloods)	1	1	3	No
**P09**	M	70	(Consulted for groin pain/swelling–hernia), abnormal blood test (‘*paraprotein spike’*).	GP (R1) Practice nurse	**R1-Haematology**	2	1	2	No
**P10**	M	74	Breathlessness, tiredness, under-performance, swollen legs, abnormal blood test (‘*kidneys shutting down’*, other abnormality–unknown what).	GP (R1)	**R1-Acute medical unit** (emergency admission via GP) • Haematology	2	2	0.5	No
**P11**	M	59	(Consulted for PSA test), frothy urine, pain (rib–later identified as fractured).	GP (R1, R2 & R3)	**R1-Urology** (biopsy, in relation to results of PSA tests) **R2-Renal medicine** (cancelled by hospital—*‘clinical decision’*) **R3-Renal medicine** • Haematology	5	3	15	Yes
P12*	M	43	Altered sensations (head/ear), enlarging lumps (head, under hair), stiffness and pain (neck), tiredness, lethargy, apathy, ‘*run-down’*, headaches; unable to open mouth/eat, under-performance.	GP (R1) Osteopath	**R1-General surgery**^5^ • Haematology	6	3	8	Yes
**P13**	M	71	Pain (ribs), breathlessness, under-performance.	GP (R1)	**R1-Oncology (Cancer of Unknown Primary)** • Haematology	3	1	7	Yes
**P14**	F	59	Pain (back–later identified as *‘vertebral collapses’*), *‘malaise’*, *‘unwell’*, height loss, nausea, pins and needles (feet), infections, loss of appetite, weight loss; abnormal blood test (unknown what–*‘positive markers’*).	Self-referral to A&E^6^ (R1) NHS Helpline to A&E (R2) GP (R3 & R4) Physiotherapist	**R1-A&E** (via self-referral) **R2-A&E** (via NHS helpline) • Medical assessment unit **R3-Acute medical unit** (emergency admission via GP) **R4-Endocrinology** (P) • Haematology	10	1	8	Yes
**P15**	M	58	Pain (back), impaired posture, reduced mobility, sleepiness; abnormal blood test (unknown what).	GP (R1) Physiotherapist	**R1-Haematology** *(‘straight away’*)	*‘Loads of times*’	1	17	Yes
**P16**	M	66	Infections, pain (ribs, chest), under-performance, abnormal blood test and scan (unknown what).	GP (R1) Physiotherapist	**R1-Oncology** (urgent) • Haematology	6	3	15	Yes
**P17**	M	70	Feeling *‘off’*, pain (back–later identified as fracture), reduced mobility, nausea, vomiting, abnormal blood test *(‘anaemia’*, *‘renal failure’*).	Self-referral to A&E (R1) GP (R2 & R3)	**R1-A&E (via self-referral)** **R2-Musculoskeletal clinic** **R3-Haematology** (urgent, then emergency admission)	5	0.5	2	No
**P18**	M	71	Weight loss, abnormal blood test *(‘anaemia’*), infection (viral–*‘flu’*), pain (back), height loss, abnormal blood test *(‘paraprotein’*).	GP (R1 & R2) Physiotherapist	**R1-Colorectal surgeon** (endoscopy and colonoscopy) **R2-Haematology** (urgent)	2	1	11	Yes
**P19**	M	62	Pain (back–later diagnosed as acute fractures), bloating, abnormal blood test *(‘protein’*), under-performance.	GP (R1) NHS helpline to A&E (R2)	**R1-Haematology (urgent)** **R2-A&E** (via NHS helpline)	2	1	1	No
**P20**	F	74	Low mood *(‘almost depressed’*), lack of energy, pain (ribs, shoulder blades, leg), headaches, weight loss, under-performance, abnormal blood tests *(‘anaemia’*).	GP Self-referral to A&E (R1)	**R1-A&E** (self-referral via ambulance) • Acute admissions unit • Orthopaedics • Haematology	4	7	5	No

^1^Based on information from interview and HMRN questionnaire

^2^**R** indicates secondary care referral and the number indicates the referral sequence

^3^bold indicates new referral; **→** indicates consultant to consultant referral

^4^Inability to perform usual activities, including: mobilising and moving (walking, bending, getting upstairs, getting into car, getting in and out of bed, driving, sitting, moving in bed, lying down, getting up); bodily actions (sneezing, coughing); hobbies/sports (golf, hiking, bell ringing, exercise bike, swimming); household jobs (gardening, washing up, moving furniture); work (lifting, general manual work)

^5^Private appointment

^6^Accident and Emergency department

*Patient also diagnosed with soft tissue plasmacytoma(s).

Interviewees described a wide range of changes in their body and health prior to diagnosis (Table 1), not all of which were initially considered symptoms of ill-health or cancer-related. Bone pain featured prominently, typically affecting the back, chest, shoulder blades, ribs or neck. This could be very severe (sometimes to the extent that physiotherapy could not be tolerated), and in some cases the pain was later attributed to fractures. Pain was also said to fluctuate at times or move from place to place: *‘it was persistent*, *and the thing was*, *it kept moving*. *It was here*, *and then it was here*, *and the pain in my back was moving’* [P05]. Fatigue was a frequent problem and often considered persistent and unusual. Pain and fatigue combined appeared to have a major impact on the ability of patients to perform their usual everyday activities (e.g. mobilising, climbing stairs, driving, household tasks and hobbies): ‘*I felt that I was worn down by the pain by that time… I just didn’t feel like doing anything ‘cause it hurt*. *Yeah*, *so you just sat still and when you’re sat still*, *you’d fall asleep’* [P02].

Health changes were commonly gradual in onset, subtle and non-specific, factors that were said to deter help-seeking: *‘yes*, *I could have been diagnosed more quickly if I’d gone to the doctors’ more quickly*, *but I didn’t realise there was anything wrong with me to go to the doctors’*, *so*, *you know I certainly can’t fault what happened… once I went’* [P05]. An overarching sense of malaise was also sometimes described, or an unwell feeling that was hard to define and explain: *‘It’s very difficult to say*. *One of the things was nausea*, *erm*, *but other than that I find it very hard to explain how I felt*, *and in the end I just ended up by saying to the doctor*, *“it’s just a feeling of malaise*. *I can’t explain how I feel”‘* [P14].

Appraisal was often complicated by the presence of comorbidities and, even in retrospect, it could be hard for patients to be sure of the extent to which their health changes were due to myeloma. Explanations were often initially found, with most patients having a ‘normalisation’ phase, in which alterations were attributed to factors such as the ageing process, a non-serious injury (e.g. pulled muscle), a new manifestation of a historical problem (e.g. bad back), or a consequence of medication: *‘It was… a gradual process*, *which I put down to old age… (and) I’d had a bad back*, *so I was quite sort of willing to accept that my back hurts a bit’* [P03]. Another common explanation was life changes or stress: ‘w*e’d got my daughter*, *son-in-law and three-year old living here… so it was very*, *very hectic here*, *and stressed’* [P02].

Some responded to their progressive symptoms by lifestyle adaptations: ‘*I gave up the (hobby) but carried on working and eventually didn’t do the heavy lifting… I could carry on doing what I had to do*, *but I stopped doing all the things that I didn’t have to do’* [P02]. One patient described an active decision to defer re-presenting to the GP due to what, in part, appeared to be fear: *‘I suppose deep down I didn’t want to know anything else*, *you know*, *we’re back to this thing that you know I think we are frightened*, *we do get frightened occasionally that… there is something more serious and so if you can sort of*, *pretend that it’s just a bad back*, *you’re quite happy to just accept that’* [P03].

A recursive process of reflection was then described whereby, in response to the severity, persistence, peculiarity or disruptiveness of changes, many patients came to question their initial appraisal, re-categorising and re-conceptualising their symptoms as abnormal: *‘I just couldn’t do anything*. *I mean I’m used to a fairly active (life)*, *even though I’m retired*, *I’ve an exercise bike in there…*, *which I used to go on every day and do 10 to 12 mile*. *When I started that I couldn’t do one mile… I knew–alarm bells–there was something wrong’* [P10]. Pain moving from place to place was often recognised as abnormal and said to promote help-seeking. Despite this, the suspicion of cancer (of any type), typically remained low: *‘cancer was miles from my mind’* [P13].

Family members were said by some interviewees to have influenced their help-seeking behaviour: ‘w*ell*, *to be honest*, *the only reason I went (to GP) was because everybody was pestering me to go’* [P02]. Occasionally, relatives acted as advocates on behalf of patients in consultations or contacted GPs independently: *‘I knew you weren’t right*, *and that’s why I ended up writing that letter (to GP)*’ [relative of P12]. In some instances, help-seeking occurred secondary to consultation for another issue: *‘the second PSA test… meant I had to go back to the GP*. *And so I told him about (the frothy urine)’* [P11]. Help was sought from a range of professionals including pharmacists, the NHS non-emergency telephone helpline (111), physiotherapists, or Accident and Emergency departments (A&E), but most initially went directly to a GP, and all eventually consulted a GP (Table 1).

Some patients felt they themselves had contributed to their late diagnosis, believing it was their responsibility to recognise the need for professional help, seek this promptly, and provide good information to practitioners: ‘*looking back*, *I misled (the GP)*, *because I assumed*, *(the malaise was) because I should never have moved in with (partner)*, *which as it happens*, *wasn’t the case… but that’s the way my mind was going*, *because (the malaise) coincided’* [P20]. Generally, interviewees also recognised the challenges of diagnosing myeloma: *‘because it’s quite a rare cancer*, *some GPs might never deal with a patient who has myeloma’* [P14].

Patient accounts often suggest that they believed their GPs did not initially tend to consider their health changes as indicative of myeloma, or something serious requiring further investigation. Interviewees often recalled GPs offering non-malignant explanations for their symptom(s), with preliminary diagnoses including chest and throat infections, oral thrush, a strain, sciatica, wry neck, restless legs, benign cysts, depression and Tietze syndrome. These diagnoses determined initial management strategies, which generally comprised reassurance, observation, treatment, and/or referral to an allied health professional. For example, where back pain was considered mechanical, analgesia and anti-inflammatories were commonly prescribed, often with advice to consult a physiotherapist or osteopath: *‘They sent you to the sports gym first of all*, *didn’t they*, *saying you’d got sciatic nerve trouble’* [relative of P03].

In the first instance, patients generally accepted the preliminary diagnoses: *‘originally*, *you know*, *I’d got confidence in his*, *in his*, *diagnosis*. *He sort of described what I was feeling’* [P02]. They often understood the GP’s rationale: ‘*I think with my symptoms and the way I presented them to (GP)*, *it was perfectly logical for him to come to the conclusion that he did’* [P02]. GP doubts regarding their preliminary diagnosis/management were considered pivotal to their reappraisal of symptoms and a change in the patient’s management. Triggers for reappraisal were patients’ re-presentation due to lack of improvement, their growing conviction that ‘*something’s wrong*’, further deterioration, or the development of new symptoms. Reappraisal often led to the instigation of investigations: *‘I think he was under the impression that what he’d given me should have worked and he was as surprised as I was when I went back*, *which is why he put me down for the endoscopy’* [P06]. Although not necessarily resulting in suspicion of myeloma, such investigations frequently identified abnormalities that prompted further tests, referral to secondary care, or emergency admission: *“Yeah*, *well*, *I think obviously they saw they were bad (blood results)*, *so they acted on them straightaway”* [P05].

Interviewees recalled between one and four hospital referrals before diagnosis (Table 1). Around a third reported at least one emergency presentation (via A&E or a direct ward admission), instigated by themselves, the NHS non-emergency telephone helpline, or their GP. Input from specialist areas (such as the high dependency unit) was also occasionally required in the time leading up to diagnosis. Where emergency presentation occurred, patients often reported symptoms indicative of advanced disease, including profoundly abnormal blood test results (e.g. suggestive of renal failure), fractured bones or severe infections (e.g. pneumonia). Few people were referred directly to haematology, and those who were tended to have an abnormal blood test result that was specific to myeloma (‘*paraprotein spike’*, ‘*protein’*, ‘*Bence Jones positive*’).

Those not referred directly to haematology described being sent to a range of specialities (Table 1), both initially and subsequently, as progress was made towards diagnosis. Referral speciality appeared to be determined by the patient’s symptoms or clinical problems; a number of referrals were made to gastroenterology/colorectal surgery (for endoscopy/colonoscopy), often in the context of anaemia with associated abdominal/bowel symptoms. Accounts suggest that in some situations cancer was suspected, but haematological malignancy was not, and this led to oncology referral. Occasionally, abnormal blood results were identified in secondary care, which resulted in patients being asked to return to hospital without a formal GP referral.

While some referrals occurred promptly, others did not appear to have been categorised as urgent and were associated with longer waits. In several instances, interviewees expedited events by requesting a private referral: *‘I rang the National Health up*, *to book an appointment*, *and the best I could do… was… October*. *So I thought*, *“Well*, *that’s no good”*, *so I thought*, *“Well*, *I’ll go private”*. *So… I tried (Private Hospital) and I got an appointment… the same week’* [P01]. On one occasion, a GP referral to the renal team resulted in a hospital appointment being issued, but then cancelled: ‘*due to a clinical decision*’ [P11].

On reaching secondary care, progress typically accelerated. Non-haematology specialists were frequently said to recognise the possibility of a haematological malignancy, conduct appropriate tests, and initiate urgent haematology referral: *‘I think she knew quite soon that these were all classic symptoms… despite that she’s not a haematologist’* [P11]. Reports about hospital care were positive; myeloma was generally identified quickly and treatment (if required) started within days/weeks of diagnosis.

### Views regarding limitations in primary care

GPs (and other primary care staff) were often perceived as lacking knowledge of myeloma, particularly regarding symptoms, relevant investigations, and the significance of test results: ‘*if the GPs were a little more au fait with the symptoms of this horrible disease*, *then perhaps we might have been referred to the correct people a lot quicker’* [relative of P14]. Whilst some participants were accepting of GPs’ limited knowledge in the context of a rare disease, others expressed certain expectations regarding practice: *‘I wouldn’t expect a GP to give a diagnosis of myeloma*, *but there are certain things that you’d anticipate’* [P12]. Gaps in history-taking were also noted, as was the absence or limited nature of physical examinations: *‘None of the GPs really*, *well*, *they didn’t measure*, *physically measure (the ‘cysts’)… they didn’t sort of press into them or anything really… they were satisfied with what they were’* [P12].

Although the need for urgent secondary care assessment was identified rapidly by certain GPs (i.e. after one or two visits), some patients reported consulting on multiple occasions before their symptoms were reappraised and hospital referral initiated: *‘if I went twice in February*, *twice March*, *twice April*, *twice June*, *erm*, *twice May*. *I don’t know*. *It’s probably getting on for about say*, *getting on for about ten times’* [P14]. Some were made to feel they were wasting the GP’s time due to repeated consultations: *‘I started to feel I was a bit of a nuisance*, *especially when the GP said to me that I was a very anxious person’* [P14].

Patients often questioned their GPs readiness to reappraise symptoms: *‘(the) doctor just kept passing it off as muscular… instead of them thinking*, *“Now hang on a minute here…” They just continued to fob you off with tablets’* [relative of P15]. As well as increasingly frequent GP visits, other common strategies to achieve reappraisal included arranging consultations with different GPs in the hope of finding an alternative perspective (‘*I went deliberately to a different one’* [P16]) and self-referral to A&E. Even when a different GP was consulted, however, the original diagnosis could be reinforced: *‘he concurred with (the first GP)*, *that they were sebaceous cysts and he never*, *nobody ever questioned the initial diagnosis and*, *you know*, *they all agreed with (first GP)’* [relative of P12].

Other patients believed that additional relevant information (e.g. further symptoms) had been dismissed by GPs, and that ‘warning signs’ (such as increased consultation rates compared to usual patterns), had been ignored: *‘I never go to the doctor’s and I was back and forth and back and forth*, *and maybe they should have picked that (up) and said*, *“Well actually*, *she never comes normally*. *Why does she keep coming back*? *Why’s she not getting any better*?*”‘* [P05]. Some patients believed investigations had taken place later than their symptoms warranted, but it was also noted that GP requests for investigations had, on occasion, been rejected in secondary care: ‘*They said I didn’t fit the required criteria (for MRI scan)*, *or my symptoms didn’t*’ [P14].

Some patients were concerned that a follow-up appointment had not been offered: *‘I feel that if it*, *if it had’ve been picked up*, *if maybe I had gone back and got my bloods done sooner… I probably wouldn’t have been so far on*. *If they’d have said*, *“Look*, *if you’re concerned*, *come back in two months and we’ll re-do your bloods”… I’d have thought a GP would have done that*, *really’* [P07]. While others were asked to return for reassessment, certain obstacles were identified to this, including: patients accepting, and being reassured by, the GP’s initial diagnosis (*‘I just sort of went along with*, *well*, *what they said*, *it were a chest infection and stuff’* [P01]); feeling they should allow adequate time for treatment to act and/or symptoms to resolve; and interpreting new symptoms in light of the initial diagnosis.

More generally, interviewees alluded to the impact of GP resources on time to diagnosis: *‘It’s the cost*, *isn’t it*? *It’s down to cost’* [P16]. Patients suggested this affected: their ability to get a GP appointment; the time allotted to them (*‘they only have the 10 minutes and so while you’re talking to them*, *they’ll be busy on the screen*, *and so they maybe just miss one crucial thing that you said’* [P03]); whether investigations were undertaken; and the likelihood that their GP could procure investigations in secondary care and timely access to specialists.

### Perceptions of changes that could improve practice

Willingness of healthcare practitioners to reappraise symptoms, and to consider all diagnostic possibilities, including malignancy, was seen as crucial to timely diagnosis. Taking precautionary measures, such as being asked to arrange a follow-up appointment, was considered important if a range of diagnoses was thought possible. Participants had varied opinions about the continuity of GP care, with some suggesting that consulting a different GP had facilitated timely diagnosis, while others perceived benefits in visiting their regular GP, as this had enabled early recognition of significant health changes compared to what was ‘normal’ for them.

## Discussion

This is the first qualitative study seeking to understand experiences in the time leading to myeloma diagnosis, using in depth analysis of patient (and relative) accounts. It describes the health changes associated with myeloma onset, how symptoms were interpreted before help-seeking, and interactions with healthcare practitioners prior to secondary care referral and diagnosis. Our findings confirm the difficulties some people experience at this time, including vague but progressive symptoms, worsening debilitation/loss of function, multiple GP consultations and emergency presentations. Accounts suggest variation in the duration of the appraisal and diagnostic intervals, and an ongoing process of assessing health changes and seeking help.

The way in which myeloma presents undoubtedly contributes to diagnostic difficulties. Our study documents a wide range of symptoms, some of which are typically associated with myeloma, such as bone pain, fatigue, fractures [10], and others that are not, such as scalp swellings, later confirmed as soft tissue plasmacytomas. It also describes the progressive underperformance experienced by many, reflected in an inability to carry out activities considered ‘normal’ for that particular individual. Many symptoms were more indicative of benign and/or self-limiting conditions than myeloma, or any other cancer, an issue compounded by problems such as bone/joint pain being typically associated with the age-group in which myeloma most often occurs, yet rarely caused by this cancer.

Such difficulties appeared to contribute to the lack of cancer suspicion among not only GPs, but also patients themselves. Myeloma is considered a cancer that is ‘harder to suspect’ in the primary care setting, as it has low predictive values for both individual and combinations of common symptoms [24]. Despite this, GPs (whilst not expected to diagnose myeloma in primary care), are still faced with the task of differentiating abnormalities that may indicate cancer from those of non-malignant conditions and making appropriate, timely referrals to secondary care.

One of the main issues causing patients to delay seeking reappraisal of their symptoms was the plausibility of the GP’s initial diagnosis and the certainty with which this was communicated. This appeared to provide a ‘lens’ through which patients subsequently assessed new symptoms, and parallels findings from a study of cancer diagnosis after emergency presentation, which found interactions with health professionals could skew patients’ re-evaluation of symptoms, leading them to postpone further help-seeking [25].

Other reasons for delay in primary care included multiple consultations before the initial diagnosis was reappraised, late reappraisal and lack of investigation and follow-up–factors found to contribute to prolonged time to diagnosis in other cancers [26,27]. While this may appear concerning, however, multiple consultations do not always indicate sub-optimal management; they may occur due to symptom monitoring, primary-care led investigations, ‘safety-netting’ (i.e. post-investigation review and follow-up of symptoms not meeting the criteria for further action, but associated with an increased risk of cancer), or patient preferences [28–30].

Although this study was conducted in the UK, where healthcare is free at the point of access via the National Health Service, a number of interviewees discussed their use of private services. This occurred either to secure prompt access to assessment and investigation in secondary care, or to address frustrations arising from what was considered sub-optimal management of health changes in primary care. Whilst some had private health insurance to cover costs, others used their own resources to purchase services.

The myriad ways in which myeloma presents suggests there may be limited value in increasing patients’ and GPs’ knowledge of the range of potential symptoms. It may be more important to encourage patients to recognize what is normal for them, and seek help when a new change in their body is detected, or when they notice progressive deterioration in their ability to perform ‘usual’ activities. For GPs, pattern recognition is important, with deviation from expected, either in terms of behaviour (e.g. consultation frequency), or symptoms, indicating that investigation and/or referral may be needed; adequate ‘safety netting’ is also crucial.

### Strengths and limitations

The main strength of this study is that it is based on in depth analysis of patient accounts of their experiences before myeloma diagnosis. To maximise recall, interviews were conducted within a year of diagnosis, relatives were invited to participate, and use of memory aides (e.g. letters, calendars and diaries) was encouraged. With the aim of providing rich narratives that encompass the breadth of experiences and promote theory building, qualitative research rarely includes wholly representative samples and seldom aspires to generalisability; thus, patients were sampled across a range of experiences and ages, and included both sexes. We did take the characteristics of the general patient population into consideration when sampling, however, so that the views of those most often affected by myeloma were captured. Our inclusion of more men than women is reflective of this, although the average diagnostic age of interviewees was somewhat lower than that of the general patient population [9]. The latter is likely to have occurred because patients were recruited from those returning a survey about symptoms and help-seeking, so the perspectives of people unable to do this (i.e. those critically ill, dying soon after diagnosis or with cognitive decline–often older patients) were not captured. There are no obvious reasons why our findings regarding symptom onset and progression would vary geographically. However, different healthcare infrastructures (such as those in countries where, unlike the UK, the delivery of services is largely provided by the private sector), may affect help-seeking and diagnostic pathways.

### Future research

GPs have considerable involvement in the pre-diagnostic care of patients later found to have myeloma, yet observational evidence exploring the extent to which patients consult, and how they are managed, is sparse. Interviews with GPs would provide the potential to learn more about the diagnostic challenges associated with complex diseases such as myeloma. In addition, the ability to routinely examine patient pathways by linking primary care databases to secondary care sources could enable analyses that are more granular and lead to improved strategies to support earlier diagnosis.
